# Supplementary material for: Bayesian and non-bayesian inference for logistic-exponential distribution using improved adaptive type-II progressively censored data
Source: PLoS One. 2024 May 16;19(5):e0298638. doi: 10.1371/journal.pone.0298638 (PMC11098395; doi:10.1371/journal.pone.0298638)
Supplement: Attachment — Submitted filename: REVIEWER REPORTS PONE.pdf [file pone.0298638.s001.pdf]

REVIEWER REPORTS

Report on: “Bayesian and Non-Bayesian inference for Logistic-exponential Distribution using Improved Adoptive Type-II Progressively Censored Data.”

Overall, I find the paper very interesting, but I think there is some room for improvement. Please, find below some comments:

1. The literature review of the different improved adoptive type-II progressively censored data is very good. I commend the authors for presenting a balanced account of the literature on adoptive type-II progressively censored data.
2. Please, indicate the motivation behind using the mechanical equipment data
3. It is not necessary to enumerate all equations. Only the ones to be referred to.
4. A word of caution regarding the introduction section, I commend the authors to split the introduction section into two different sections, section I entitled “Introduction” and another section entitled “model formulation” to avoid misguiding
5. The baseline model proposed in this study is ***logistic-exponential***. I ***strongly*** recommend discussing about what motivates to use this baseline compared to other existing ones.
6. In general, I encourage the authors to reflect on whether the included material goes beyond a marginal contribution that perhaps could be find on textbooks instead. This will help them make a more careful selection of the material included in the paper, and to present a more concise message.
7. Overall, I think the paper could be shorten to about 15 pages without affecting the main contributions.
8. A detailed subsection about the convergence diagnostics is recommendable
9. A proofread is recommended.

Submission Date

18th December 2023
