# Supplementary material for: Bayesian and non-bayesian inference for logistic-exponential distribution using improved adaptive type-II progressively censored data
Source: PLoS One. 2024 May 16;19(5):e0298638. doi: 10.1371/journal.pone.0298638 (PMC11098395; doi:10.1371/journal.pone.0298638)
Supplement: Attachment — Submitted filename: Response_to_Reviewers.pdf [file pone.0298638.s002.pdf]

Response letter:

"Bayesian and Non-Bayesian inference for Logistic-exponential Distribution using Improved Adoptive Type-II Progressively Censored Data.

December 26, 2023

We sincerely appreciate your insightful comments on the model formulation and the detailed suggestions for improving the clarity of the results section. We are committed to incorporating your feedback to strengthen the paper and address your valuable questions.

Reviewer 1

Comment 1: *The literature review of the different improved adoptive type-II progressively censored data is very good. I commend the authors for presenting a balanced account of the literature on adoptive type-II progressively censored data.*

Response: We are sincerely grateful for your positive feedback on our literature review. We value your perspective on the balanced account of adoptive type-II progressively censored (AT-II PC) data. As you suggested, we have added some literature regarding the AT-II PCS. (See page 2)

Comment 2: *Please, indicate the motivation behind using the mechanical equipment data.*

Response: Thank you for your valuable question. This data set fits LE distribution better than other existing distribution as Weibull distribution. further, the data set shows the applicability of proposed methods in engineering domain.

Comment 3: *It is not necessary to enumerate all equations. Only the ones to be referred to.*

Response: Thank you for your careful reading and valuable suggestion. According to your suggestion, all equations are not enumerated in the revised manuscript.

Comment 4: *A word of caution regarding the introduction section, I commend the authors to split the introduction section into two different sections, section I entitled "Introduction" and another section entitled "model formulation" to avoid misguiding.*

Response: Thank you for your valuable suggestion. According to your suggestion, introduction has been splitted into two sections in the revised manuscript. (See pages 1-4)

Comment 5: *The baseline model proposed in this study is logistic-exponential. I strongly recommend discussing about what motivates to use this baseline compared to other existing ones.*

Response: Thank you for your valuable concern. According to your suggestion, the applicability of LED has been incorporated in the revised manuscript. (See page 3)

Comment 6: *In general, I encourage the authors to reflect on whether the included material goes beyond a marginal contribution that perhaps could be find on textbooks instead. This will help them make a more careful selection of the material included in the paper, and to present a more concise*

message.

Response: Thank you for your careful reading and valuable suggestion. In the revised manuscript we have omitted and added the required material accordingto your suggestion.

Comment 7: *Overall, I think the paper could be shorten to about 15 pages without affecting the main contributions.*

Response: Thank you for your suggestion. According to your suggestion, we tried to revise and shorten this within 16 pages without affecting the main contributions.

Comment 8: *A detailed subsection about the convergence diagnostics is recommendable.*

Response: Thank you for your valuable suggestion. For the convergence of the MCMC samples we have already depicted the trace plots of the samples which denotes the values of the samples are convergent. (See page 13)

Comment 9: *A proofread is recommended.*

Response: Thank you for your careful reading and invaluable suggestion. According to your suggestion, we have gone through the manuscript very carefully and omit the errors as much as possible.

Reviewer 2:

Comment 1: *What were the main findings or insights you gained from the numerical analysis? Were there any unexpected or counter intuitive results?*

Response: Thank you for your careful reading and valuable suggestion. According to your suggestion the main insights of the smulation study has been incorporated in the revised manuscript. (See page 10). Further, any type of unexpected or counter intuitive results was not found in this simulation study.

Comment 2: *How did you select the two actual data sets for demonstration purposes?*

Response: Thank you for your insightful comment. In the real data analysis, it has been found that this mechanical equipment data set fits the logistic exponential distribution better than the existing result associated with Weibull distribution.

Comment 3: *What were the characteristics of these data sets, and how did they relate to the theoretical results of the study?*

Response: Thank you for such valuable comment. This data set is a better fit with the logistic exponential distribution. From the real data analysis, it has been noticed that the proposed estimates behave similar for the estimates based on this data.

Comment 4: *What are some future directions for research in this area?*

Response: Thank you for your valuable comment. The future direction has been included in the revised manuscript, according to your suggestion. (See page 14)

Comment 5: *Are there any other estimation methods or entropy measures that could be explored?*

Response: Thank you for such insightful comment. It is possible to extend this work for entropy estimation along with adding another classical estimation method such as maximum product spacing.

Comment 6: *How can the confidence intervals of the entropies be estimated using various estimation methods?*

Response: Thank you for your comment. We have not been dealing with entropy in this manuscript. But we are dealing with reliability characteristics and for that we have utilized delta method to obtain the confidence intervals.

Comment 7: *There is a need of some more literature regarding the Adaptive Type-II Progressive Censored and the different estimation procedures of distribution. The author may use some references.*

Response: Thank you for your insightful suggestion! According to your suggestion, we have incorporated some of the references related to AT-II PCS in the revised manuscript. (See page 2)

Comment 8: *Discuss the behavior of graphs in detail.*

Response: Thank you for your valuable comment.

Comment 9: *How do we distinguish that the parameters of MLE are not a local maximum?*

Response: Thank you for your insightful comment. To show the uniqueness and existence of the MLEs in a graphical approach, a contour plot has been incorporated in the revised manuscript. This plot distinguish that the parameters of MLE are not a local maximum. (See Page 14)

Comment 10: *The authors should explain how the p-values for the KS test have been calculated. This is not a straightforward problem in situations when model parameters have to be estimated.*

Response: Thank you for your insightful comment. In this case we have calculated the p-values for the complete data set based on the MLEs which are already mentioned in the manuscript.

Comment 11: *The authors use MCMC to estimate the posterior density function. However, there are other estimators such as KDE with tighter convergence rates that they could explore.*

Response: Thank you for your valuable suggestion. In this study we focuses on parametric estimation only. We may extend the KDE estimator which is one of the non-parametric estimator in some other problem.

Comment 12: *Take care of the formatting of the references.*

Response: Thank you for you valuable comment. All the references are formatted accordingly the suggestion.

Comment 13: *In the references section, it is necessary to remove the reference that the author doesn't use in the manuscript. Please check.*

Response: Thank you for such careful reading and valuable comment. All the unnecessary referenecs are removed in the revised manuscript.
