# Supplementary material for: Bayesian and non-bayesian inference for logistic-exponential distribution using improved adaptive type-II progressively censored data
Source: PLoS One. 2024 May 16;19(5):e0298638. doi: 10.1371/journal.pone.0298638 (PMC11098395; doi:10.1371/journal.pone.0298638)
Supplement: Attachment — Submitted filename: Response_to_Reviewers__IAT_II_PCS_LE_.pdf [file pone.0298638.s004.pdf]

Response letter:

"Bayesian and Non-Bayesian inference for Logistic-exponential Distribution using Improved Adoptive Type-II Progressively Censored Data.

January 14, 2024

We sincerely appreciate your insightful comments on the model formulation and the detailed suggestions for improving the clarity of the results section. We are committed to incorporating your feedback to strengthen the paper and address your valuable questions.

Reviewer 3:
Comment 1: *In section 1, the authors used the Logistic-exponential distribution as a lifetime distribution, please write the authors who introduced this distribution. Define the type of each parameter. Add plots of the density and hazard rate functions according to the descriptions mentioned after equation 1.4.*

Response: Thank you for your careful reading and valuable suggestion. The author names have been mentioned in the introduction section in the revised manuscript. Further, the figures of PDF and HRF are also incorporated in the revised manuscript. (See page 2)

Comment 2: *In section 1, at the end of second paragraph, this sentence "This work specifically examines censored samples using an enhanced adaptive Type-II progressive censoring method. The objective is to estimate the unknown parameters of the LED" has no meaning there, please remove it.*

Response: Thank you for your careful reading. That line has been removed in the revised manuscript according to your suggestion.

Comment 3: *In log-likelihood function equation this term* $\sum_{i=1}^{E_2} ln(\Psi_{x_i} + 1)$ *must be corrected to* $\sum_{i=1}^{E_2} \nu x_i$*, and must be corrected in equation (3.5) to* $\sum_{i=1}^{E_2} x_i$

Response: Thank you for your valuable suggestion. According to your suggestion, all these terms are rectified in the revised manuscript.

Comment 4: *Also this term* $R_i \sum_{i=1}^{E_1} ln(1 + \Psi_{x_i}^{\eta})$ *must be corrected to, also this term* $\sum_{i=1}^{E_1} R_i ln(1 + \Psi_{x_i}^{\eta})$ *must be corrected in all subsequent equations (3.3), (3.4),(3.5).*

Response: Thank you for your valuable suggestion. According to your suggestion, all these equations are modified in the revised manuscript. (See page 5)

Comment 5: *According to the previous corrections all the second derivatives must be checked carefully? Also, check all equations carefully.*

Response: Thank you for your valuable suggestion. According to your suggestion, all these equations have been checked once thoroughly.

Comment 6: *In equation (3.7),* $-\frac{\partial^2 l(\nu,\eta|\mathbf{x})}{\partial\nu\eta}, -\frac{\partial^2 l(\nu,\eta|\mathbf{x})}{\partial\eta\nu}$ *must be corrected to* $-\frac{\partial^2 l(\nu,\eta|\mathbf{x})}{\partial\nu\partial\eta}, -\frac{\partial^2 l(\nu,\eta|\mathbf{x})}{\partial\eta\partial\nu}$ *Also, the same correction must be made for the observed FIM.*

Response: Thank you for your valuable suggestion. According to your suggestion, all these terms are modified in the revised manuscript. (See page 6)

Comment 7: *At the end of page 7, approximate confidence intervals (ACIs) must be written as ACIs*

Response: Thank you for your valuable suggestion. According to your suggestion, that is modified in the revised manuscript.

Comment 8: *After equation (4.1), SELF, and LLF must be defined. After algorithm 1 SEL and LL must be defined.*

Response: Thank you for your valuable suggestion. According to your suggestion, SELF and LLF are defined in the revised manuscript. (See page 8)

Comment 9: *The selected value of p in linear exponential loss function must be added in section 5.*

Response: Thank you for your careful reading. The value of p has been incorporated in section 5 of the revised manuscript. (See page 10)

Comment 10: *The visibility and clarity of all tables must be enhanced. In table 5, (SEs in parenthesis), SEs must be defined*

Response: Thank you for your valuable suggestion. We have tried to increase the clarity of all tables in the revised manuscript. In table 5, SE is defined as standard error in the revised manuscript.

Comment 11: *Why do the authors consider just this only one true value of parameter in simulation study? Do they correspond to any experience from practice?*

Response: Thank you for your valuable comment. We have taken one set of value of the parameters due to make less number of tables, even we have been suggested to reduce the pages too. We have checked for other sets of parameters values too which is giving similar results.

Comment 12: *The conclusions should be added by discussing limitations of the proposed methods?*

Response: Thank you for your valuable suggestion. A limitation of this study which is found at the time of simulation, has been incorporated in the conclusion of the revised manuscript. (See page 15)

Some corrections

Comment 13: *Cohen and Clifford [1] must be corrected to Cohen [1]*

Response: Thank you for your careful reading. The suggested correction has been made in the revised manuscript. (See page 1)

Comment 14: *In introduction, this abbreviation PDF and CDF must be defined.*

Response: Thank you for your careful reading and valuable suggestion. These have been defined in the revised manuscript. (See page 2)

Comment 15: *In introduction section, LE distribution must be written as LED, third line of last paragraph in introduction section LE replaced with LED. In all article check the abbreviation LE?*

Response: Thank you for your careful reading and suggestion. All these have been modified in the revised manuscript.

Comment 16: *At the end of page 3, the authors write pdf and cdf, write this abbreviation in only one form, pdf, cdf or PDF, CDF.*

Response: Thank you for your valuable comment. All these abbreviation have been made in one form in the revised manuscript.

Reviewer 1

Comment 1: *The literature review of the different improved adoptive type-II progressively censored data is very good. I commend the authors for presenting a balanced account of the literature on adoptive type-II progressively censored data.*

Response: We are sincerely grateful for your positive feedback on our literature review. We value your perspective on the balanced account of adoptive type-II progressively censored (AT-II PC) data. As you suggested, we have added some literature regarding the AT-II PCS. (See page 2)

Comment 2: *Please, indicate the motivation behind using the mechanical equipment data.*

Response: Thank you for your valuable question. This data set fits LE distribution better than other existing distribution as Weibull distribution. further, the data set shows the applicability of proposed methods in engineering domain.

Comment 3: *It is not necessary to enumerate all equations. Only the ones to be referred to.*

Response: Thank you for your careful reading and valuable suggestion. According to your suggestion, all equations are not enumerated in the revised manuscript.

Comment 4: *A word of caution regarding the introduction section, I commend the authors to split the introduction section into two different sections, section I entitled "Introduction" and another section entitled "model formulation" to avoid misguiding.*

Response: Thank you for your valuable suggestion. According to your suggestion, introduction has been splitted into two sections in the revised manuscript. (See pages 1-4)

Comment 5: *The baseline model proposed in this study is logistic-exponential. I strongly recommend discussing about what motivates to use this baseline compared to other existing ones.*

Response: Thank you for your valuable concern. According to your suggestion, the applicability of LED has been incorporated in the revised manuscript. (See page 3)

Comment 6: *In general, I encourage the authors to reflect on whether the included material goes beyond a marginal contribution that perhaps could be find on textbooks instead. This will help them make a more careful selection of the material included in the paper, and to present a more concise message.*

Response: Thank you for your careful reading and valuable suggestion. In the revised manuscript we have omitted and added the required material accordingto your suggestion.

Comment 7: *Overall, I think the paper could be shorten to about 15 pages without affecting the main contributions.*

Response: Thank you for your suggestion. According to your suggestion, we tried to revise and shorten this within 16 pages without affecting the main contributions.

Comment 8: *A detailed subsection about the convergence diagnostics is recommendable.*

Response: Thank you for your valuable suggestion. For the convergence of the MCMC samples we have already depicted the trace plots of the samples which denotes the values of the samples are convergent. (See page 13)

Comment 9: *A proofread is recommended.*

Response: Thank you for your careful reading and invaluable suggestion. According to your suggestion, we have gone through the manuscript very carefully and omit the errors as much as possible.

Reviewer 2:

Comment 1: *What were the main findings or insights you gained from the numerical analysis? Were there any unexpected or counter intuitive results?*

Response: Thank you for your careful reading and valuable suggestion. According to your suggestion the main insights of the smulation study has been incorporated in the revised manuscript. (See page 10). Further, any type of unexpected or counter intuitive results was not found in this simulation study.

Comment 2: *How did you select the two actual data sets for demonstration purposes?*

Response: Thank you for your insightful comment. In the real data analysis, it has been found that this mechanical equipment data set fits the logistic exponential distribution better than the existing result associated with Weibull distribution.

Comment 3: *What were the characteristics of these data sets, and how did they relate to the theoretical results of the study?*

Response: Thank you for such valuable comment. This data set is a better fit with the logistic exponential distribution. From the real data analysis, it has been noticed that the proposed estimates behave similar for the estimates based on this data.

Comment 4: *What are some future directions for research in this area?*

Response: Thank you for your valuable comment. The future direction has been included in the revised manuscript, according to your suggestion. (See page 14)

Comment 5: *Are there any other estimation methods or entropy measures that could be explored?*

Response: Thank you for such insightful comment. It is possible to extend this work for entropy estimation along with adding another classical estimation method such as maximum product spacing.

Comment 6: *How can the confidence intervals of the entropies be estimated using various estimation methods?*

Response: Thank you for your comment. We have not been dealing with entropy in this manuscript. But we are dealing with reliability characteristics and for that we have utilized delta method to obtain the confidence intervals.

Comment 7: *There is a need of some more literature regarding the Adaptive Type-II Progressive Censored and the different estimation procedures of distribution. The author may use some references.*

Response: Thank you for your insightful suggestion! According to your suggestion, we have incorporated some of the references related to AT-II PCS in the revised manuscript. (See page 2)

Comment 8: *Discuss the behavior of graphs in detail.*

Response: Thank you for your valuable comment.

Comment 9: *How do we distinguish that the parameters of MLE are not a local maximum?*

Response: Thank you for your insightful comment. To show the uniqueness and existence of the MLEs in a graphical approach, a contour plot has been incorporated in the revised manuscript. This plot distinguish that the parameters of MLE are not a local maximum. (See Page 14)

Comment 10: *The authors should explain how the p-values for the KS test have been calculated. This is not a straightforward problem in situations when model parameters have to be estimated.*

Response: Thank you for your insightful comment. In this case we have calculated the p-values for the complete data set based on the MLEs which are already mentioned in the manuscript.

Comment 11: *The authors use MCMC to estimate the posterior density function. However, there are other estimators such as KDE with tighter convergence rates that they could explore.*

Response: Thank you for your valuable suggestion. In this study we focuses on parametric estimation only. We may extend the KDE estimator which is one of the non-parametric estimator in some other problem.

Comment 12: *Take care of the formatting of the references.*

Response: Thank you for you valuable comment. All the references are formatted accordingly the suggestion.

Comment 13: *In the references section, it is necessary to remove the reference that the author doesn't use in the manuscript. Please check.*

Response: Thank you for such careful reading and valuable comment. All the unnecessary referenecs are removed in the revised manuscript.
