# Supplementary material for: Bayesian and non-bayesian inference for logistic-exponential distribution using improved adaptive type-II progressively censored data
Source: PLoS One. 2024 May 16;19(5):e0298638. doi: 10.1371/journal.pone.0298638 (PMC11098395; doi:10.1371/journal.pone.0298638)
Supplement: Attachment — Submitted filename: Reviewer comments.pdf [file pone.0298638.s003.pdf]

Date 6-1-2024

Referee's Report

Journal: PLOS ONE

Title "**Bayesian and Non-Bayesian inference for Logistic-exponential Distribution using Improved Adaptive Type-II Progressively Censored Data**"

Manuscript number PONE-D-23-40731R1

My Comments

1. In section 1, the authors used the Logistic-exponential distribution as a lifetime distribution, please write the authors who introduced this distribution. Define the type of each parameter. Add plots of the density and hazard rate functions according to the descriptions mentioned after equation 1.4.
2. In section 1, at the end of second paragraph, this sentence "This work specifically examines censored samples using an enhanced adaptive Type-II progressive censoring method. The objective is to estimate the unknown parameters of the LED" has no meaning there, please remove it.
3. In log-likelihood function equation this term $\sum_{i=1}^{E_2} \ln(\Psi_{x_i} + 1)$ must be corrected to $\sum_{i=1}^{E_2} v x_i$, and must be corrected in equation (3.5) to $\sum_{i=1}^{E_2} x_i$
4. Also this term $R_i \sum_{i=1}^{E_1} \ln(1 + \Psi_{x_i}^{\eta})$ must be corrected to, $\sum_{i=1}^{E_1} R_i \ln(1 + \Psi_{x_i}^{\eta})$ also this term must be corrected in all subsequent equations (3.3), (3.4),(3.5).
5. According to the previous corrections all the second derivatives must be checked carefully? Also, check all equations carefully.
6. In equation (3.7), $\frac{-\partial^2 l(v,\eta|x)}{\partial \eta v}, \frac{-\partial^2 l(v,\eta|x)}{\partial v \eta}$ must be corrected to $\frac{-\partial^2 l(v,\eta|x)}{\partial \eta \partial v}, \frac{-\partial^2 l(v,\eta|x)}{\partial v \partial \eta}$. Also, the same correction must be made for the observed FIM.
7. At the end of page 7, approximate confidence intervals (ACIs) must be written as ACIs
8. After equation (4.1), SELF, and LLF must be defined. After algorithm 1 SEL and LL must be defined.
9. The selected value of p in linear exponential loss function must be added in section 5.
10. The visibility and clarity of all tables must be enhanced. In table 5, (SEs in parenthesis), SEs must be defined
11. Why do the authors consider just this only one true value of parameter in simulation study? Do they correspond to any experience from practice?
12. The conclusions should be added by discussing limitations of the proposed methods?

Some corrections

13. Cohen and Clifford [1] must be corrected to Cohen [1]
14. In introduction, this abbreviation PDF and CDF must be defined.
15. In introduction section, LE distribution must be written as LED, third line of last paragraph in introduction section LE replaced with LED. In all article check the abbreviation LE?
16. At the end of page 3, the authors write pdf and cdf, write this abbreviation in only one form, pdf, cdf or PDF, CDF.
